# Predictors of RBD progression and conversion to synucleinopathies

**DOI:** 10.1007/s11910-022-01171-0

**Published:** 2022-03-11

**Authors:** Edoardo Rosario de Natale, Heather Wilson, Marios Politis

**Affiliations:** Neurodegeneration Imaging Group, University of Exeter Medical School, London, UK

**Keywords:** Rapid eye movement sleep behaviour disorder, Synucleinopathies, Biomarkers

## Abstract

**Purpose of review:**

Rapid eye movement (REM) sleep behaviour disorder (RBD) is considered the expression of the initial neurodegenerative process underlying synucleinopathies and constitutes the most important marker of their prodromal phase. This article reviews recent research from longitudinal research studies in isolated RBD (iRBD) aiming to describe the most promising progression biomarkers of iRBD and to delineate the current knowledge on the level of prediction of future outcome in iRBD patients at diagnosis.

**Recent findings:**

Longitudinal studies revealed the potential value of a variety of biomarkers, including clinical markers of motor, autonomic, cognitive, and olfactory symptoms, neurophysiological markers such as REM sleep without atonia and electroencephalography, genetic and epigenetic markers, cerebrospinal fluid and serum markers, and neuroimaging markers to track the progression and predict phenoconversion. To-date the most promising neuroimaging biomarker in iRBD to aid the prediction of phenoconversion is striatal presynaptic striatal dopaminergic dysfunction.

**Summary:**

There is a variety of potential biomarkers for monitoring disease progression and predicting iRBD conversion into synucleinopathies. A combined multimodal biomarker model could offer a more sensitive and specific tool. Further longitudinal studies are warranted to iRBD as a high-risk population for early neuroprotective interventions and disease-modifying therapies.

## Introduction

Rapid eye movement (REM) sleep behaviour disorder (RBD) is a parasomnia clinically characterized by active dream enactment, including screaming, flinging, or falling off from bed, that may cause injuries to patients and their bed partners [1]. According to the American Academy of Sleep Medicine, the diagnosis of RBD relies on the confirmation, with video-polysomnography (PSG), of decreased muscle atonia and sudden movements during sleep [2]. RBD is considered a common disorder. The pooled prevalence of PSG-confirmed RBD has been estimated at 0.68% of the general population, and that of probable RBD at 5.65% [3].

Isolated RBD (iRBD) is now considered the expression of the initial neurodegenerative process underlying synucleinopathies, including Parkinson’s disease (PD), Dementia with Lewy Bodies (DLB) and Multiple System Atrophy (MSA) [4] and constitutes the most important marker of the prodromal phase of these neurodegenerative diseases [5]. Large longitudinal cohort studies have demonstrated that 81–91% of iRBD patients, followed-up for at least 14 years, will develop either a definite neurodegenerative disease or a mild cognitive impairment [6, 7].

iRBD is considered a marker of neurodegeneration with strong predictive value and scarce sensitivity. The likelihood ratio of PSG-proven iRBD for development of a synucleinopathy has been estimated at 130, more than three times higher than that of detecting striatal dopamine loss on molecular imaging [8]. By contrast, only about 50% of all PD patients have experienced iRBD in their prodromal stage [9].

In the future, iRBD could be considered a condition which is useful to be targeted with new disease-modifying therapies, currently in development aiming to interrupt the pathological processes towards the development of synucleinopathies at an earlier stage. The study of iRBD pathology can allow the understanding of biological alterations preceding the clinical manifestation of a synucleinopathy, thus anticipating personalized treatments at a stage where cellular damage could be reversible [10]. To do so, we need to understand which characteristics of iRBD are associated with pathological progression, and with faster or slower phenoconversion [11]. The past few years have seen an increase of longitudinal studies aimed at establishing the value of multiple clinical, genetic, neurophysiological, fluid, and imaging biomarkers in the prediction of the progression from iRBD to PD, DLB, and MSA [12].

This article reviews the most relevant results from recent longitudinal research studies in iRBD, with the intent of describing the most promising progression biomarkers of iRBD and to delineate the current knowledge on the level of prediction in iRBD patients at diagnosis and their future outcome (Table 1).

**Table 1:** List of the most important predictors of progression of isolated RBD towards synucleinopathy

Area	Biomarker	Effect	References
Clinical	UPDRS-III	Early appearance of speech and voice alterations, followed by bradykinesia, rigidity and rest tremor. Faster progression in PD converters	[13••, 14••]
	Quantitative autonomic scales scores	Urinary symptoms scores more severe in MSA converters, decline in systolic blood pressure more pronounced in DLB converters	[13••, 15]
	Heart Rate Variability	Decreased in iRBD patients. Conflicting results on its predictive value	[16, 17, 18]
	Cognitive dysfunction	Alterations in attention, executive function, and verbal memory associated with faster conversion in iRBD. DLB converters show more pronounced cognitive alterations at baseline and faster progression	[13••, 19, 20, 21]
	Hyposmia	Associated with higher risk of phenoconversion in iRBD to DLB and PD, but not MSA	[13••, 22, 23, 24, 25]
	Visual dysfunction	Abnormal colour vision associated with increased risk of phenoconversion. Faster progression in DLB converters	[14••, 26]
Genetic	*GBA* gene variants	Higher rate of GBA variants in iRBD patient with higher rate of phenoconversion. Risk influenced by the severity of the mutation	[27, 28, 29••, 30]
	*TMEM175* gene mutations	The p.Q65P variant associated with increased rate of phenoconversion to a synucleinopathy	[31••]
	*SNCA* gene CpG hypomethylation	Associated with increased risk of progression of iRBD symptoms and phenoconversion	[32•]
Neurophysiological	RSWA	Percentage of RSWA at baseline is a predictor of future phenoconversion. Tonic RSWA associated with higher risk of future conversion to parkinsonism, phasic RSWA associated with risk of future phenoconversion to dementia	[33, 34•, 35]
	Isolated RSWA	Conflicting results on its predicting role of future phenoconversion	[36, 37, 38]
	EEG abnormalities	Higher δ and θ power in the cortex and higher slow-to-fast power ratio in converters. Diffuse slowing of electrical activity associated with DLB; EEG slowing in temporal and occipital lobes associated with PD	[39, 40]
	Cyclic Alternating Pattern (CAP)	CAP rate reduction in future converters to a neurodegenerative disease	[41]
Fluid	p-tau/total tau ratio in CSF	Reduced ratio in iRBD associated with phenoconversion at five years	[31••]
Imaging	Presynaptic striatal dopamine terminals [^123^I]FP-CIT SPECT and [^99m^TC]TRODAT-1 SPECT	Progressive loss of presynapric dopamine terminals in the striatum associated with high risk of short-term conversion	[42, 43, 44, 45]
	Glucose metabolism [^99m^Tc]ECD SPECT and [^18^F]FDG PET	Increased hippocampal perfusion in iRBD who phenoconvert at three years. Alterations in PD-related metabolic pattern associated with phenoconversion in iRBD	[46, 47, 48••]
	Structural MRI	Cortical thinning in frontal, parietal, and occipital cortices associated with phenoconversion in iRBD	[31••, 49••]

Abbreviations: CSF: Cerebrospinal fluid; DLB: Dementia with Lewy bodies; EEG: Electroencephalogram; GBA: glucocerebrosidase; MRI: Magnetic resonance imaging; MSA: Multiple system atrophy; PD: Parkinson’s disease; PET: Positron emission tomography; RBD: REM Sleep behaviour disorder; RSWA: REM sleep without atonia; SNCA: Synuclein; SPECT: Single photon emission computerized tomography; TMEM175: Transmembrane protein 175; UPDRS: Unified Parkinson’s disease rating scale

## Clinical Markers of Progression

Prodromal PD is a clinical entity characterized by the presence of motor and non-motor symptoms, such as alterations in motor dexterity, autonomic dysfunction, mood, olfaction, and cognition, that reflect the progressing neuronal damage in the brain [50]. Many of these clinical symptoms co-occur in iRBD and have been extensively studied in large, multicentre, longitudinal studies to assess whether they could represent a sign of progressing degeneration and a predictor of short-term diagnosis of a neurodegenerative disease.

### Motor

An akinetic-rigid syndrome is a hallmark feature of synucleinopathies and current dopaminergic therapy is principally directed at improving these symptoms. In future PD converters with iRBD, performance on tasks assessing motor dexterity can be altered as early as 12.9 years from diagnosis [13••]. Additionally, an increase in the Unified Parkinson’s disease Rating Scale part III (UPDRS-III) score is first detected at around 6.5 years before diagnosis and accelerates in the final 1–2 years before that [13••]. The increase of UPDRS-III score is initially driven by speech and voice alterations, followed in time by bradykinesia, rigidity and, lastly, rest tremor [13••]. In a large multicentric study performed by Postuma and colleagues, the hazard ratio of both UPDRS-III score and of performance on quantitative motor tests was comparable, in entity, to that of altered striatal uptake on [^123^I]FP-CIT SPECT, a marker of presynaptic dopamine transporter (DAT) availability [14••]. In iRBD patients converting to DLB, motor symptoms appear earlier than in PD converters but, differently from the latter, they progress at a slower pace [14••]. Overall, alterations in motor performances yield similar degrees of prediction towards future development of either dementia or parkinsonism [14••].

### Autonomic

Up to 94% of iRBD patients report symptoms of autonomic dysfunction [51]. Studies of autonomic function in iRBD have employed specific scales and questionnaires (such as Scales for Outcomes in PD—Autonomic Dysfunction (SCOPA-AUT) and the Non-Motor Symptoms Questionnaire (NMSQ)) as well as instrumental tests (heart rate variability, cardiovascular reflex testing, cardiac scintigraphy, etc.) to assess autonomic alteration [52]. Symptoms due to sympathetic dysfunction in iRBD can be detected through administration of clinical scales as early as 16 years before clinical diagnosis and total scores of these scales become statistically different from controls at around 4–6 years from diagnosis [13••].

High scores on specific autonomic symptoms are found more frequently in iRBD converting to a specific synucleinopathy. MSA converters show more severe urinary symptoms, whereas DLB converters show faster declines of systolic blood pressure. In a recent small study on 18 iRBD patients, in which the severity of alterations of preganglionic and postganglionic sudomotor, cardiovagal, and cardiovascular adrenergic function on instrumental tests was converted to a composite score (CASS score), it was found that the iRBD who converted to DLB had a longer duration of autonomic dysfunction and a higher degree of impairment of cardiovagal and, to a lesser degree, adrenergic autonomic dysfunction, compared to those who converted to PD [15].

A decreased heart rate variability (HRV) recorded on full-night PSG is an early feature of prodromal PD [53] and is also found in iRBD patients [16]. Alterations in beat-to-beat variability in a cohort of iRBD patients studied longitudinally for an average 6.7 years, however, did not discriminate between patients who eventually converted from those who did not [17]. Very recently, presence of low HRV in a cohort of 47 iRBD was associated with severity of the quantified tonic REM Sleep without Atonia (RSWA), an electrophysiological marker of severity of RBD and possible predictor of phenoconversion [18].

### Cognitive Dysfunction

iRBD patients frequently display cognitive dysfunction [54] which progresses over time [55, 56, 57]. In turn, about 35% of patients with Mild Cognitive Impairment (MCI) have iRBD [58]. The most affected cognitive domains in iRBD are attention, executive functions, and visuospatial abilities [19–21, 58••, 59, 60••]. Dysfunction of these cognitive abilities in iRBD patients has been associated with faster conversion to a neurodegenerative disease in studies with short follow-up (fewer than three years) [19, 21]. On the basis of clinical, neuroimaging, and neurophysiological findings, it has also been suggested that iRBD plus MCI may represent a distinct, more aggressive phenotype that iRBD alone [21, 60••, 61, 62, 63].

A number of recent studies have investigated the cognitive profile, and the trajectory of cognitive impairment progression in iRBD patients who eventually convert to DLB, as opposed to PD. DLB converters examined up to six years before diagnosis, already show alterations on attention, executive function, and verbal memory with subsequent development of deficits in episodic verbal learning and memory and a faster progression compared to PD converters [13••, 19, 20]. PD converters, by contrast, display cognitive performances within normal limits until 1–2 years before diagnosis [19]. Overall, presence at baseline of multidomain cognitive dysfunction in iRBD patients is the main clinical characteristics able to predict whether a patient will end up developing DLB or PD [13••].

### Hyposmia

Olfactory impairment is a frequent symptom in iRBD [64]. Odour identification tests have been widely employed in clinical studies to evaluate olfactory impairment in iRBD patients. Alterations in odour identification scores can be spotted, in iRBD patients, as early as 22 years before diagnosis of a neurodegenerative condition and become significantly impaired compared with controls nine years before phenoconversion [13••]. Hyposmia in iRBD, however, does not seem to progress at a faster pace than in normal ageing [13••, 22].

Alterations of odour identification has been linked with a 7.3-fold increased risk of developing a synucleinopathy within five years [24]. These results have been replicated in a recent larger study of 140 iRBD patients that underwent odour identification test and were followed up for an average 5.6 years. Here, hyposmia was associated to a higher risk of developing either PD or DLB in the short term, without however discriminating prospectively between the two conditions [22].

Studies on MSA converters are hindered by the small sample sizes but suggest that olfactory dysfunction at baseline does not predict the future conversion to MSA. In one study of twelve iRBD patients tested four years before conversion to MSA, hyposmia was present in 50%, a percentage higher than controls but significantly lower than in PD [25]. In the study by Iranzo and colleagues, the three iRBD patients eventually diagnosed with MSA after follow-up were all normosmic [22]. These findings are consistent with the low prevalence of olfactory dysfunction in the clinical picture of MSA [23].

### Visual Dysfunction

Patients with iRBD exhibit different degrees of visual dysfunction. These encompass abnormal colour discrimination and stereopsis and illusions [65]. Studies on visual dysfunction have employed a range of tests, from contrast sensitivity tests to colour vision discrimination tests. Abnormal colour vision in iRBD is associated with a higher risk of developing a neurodegenerative synucleinopathy [26]. Colour vision testing performed at baseline can identify iRBD patients who will later convert to DLB as opposed to those who will later convert to PD [14••]. In addition, the trajectory of colour vision impairment in DLB converters progression is steeper than that of PD converters [14••]. However, the clinical test used to assess colour vision discrimination has a visuoperceptual cognitive component that may bias this result [66].

In a recent small study, the visual acuity and the contrast sensitivity of 12 iRBD has been found to be reduced compared to controls, and further declined after a one-year follow-up [67]. Further tests would be needed to assess whether this could constitute a possible marker of progression in iRBD.

## Genetic Markers of Progression

Around 5–10% of all PD cases can be ascribed to single gene mutations. In the last twenty years, several rare, highly-penetrant mutations with Mendelian inheritance, as well as frequent variants with smaller effects, have been discovered [68]. Mutations of the *GBA* gene, encoding for Glucocerebrosidase, are associated with higher risk of PD and DLB [69, 70, 30]. In these cases, the frequency of RBD is higher than in non-*GBA* cases, and the severity of the phenotype is influenced by the type of the *GBA* mutation [71]. Patients with iRBD display higher frequency of *GBA* mutations compared to healthy controls, and comparable to that of PD patients [27, 72, 73, 28]. Within iRBD patients, *GBA* mutation carriers tend to have an earlier age at onset, but do not present any other distinctive phenotypic characteristics compared to iRBD patients negative for *GBA* mutations [28].

Three recent studies have attempted to establish whether *GBA* mutations in iRBD confer with higher risk of phenoconversion. In one study with 8 iRBD *GBA* carriers, no such association was detected [27]. In another study with 13 iRBD with *GBA* mutations, a 3.2-fold higher rate of phenoconversion towards parkinsonism and/or dementia was detected [28]. In 2020, Krohn and colleagues gathered a large multicentre longitudinal database of 1061 patients with iRBD, of which 9.5% carried a *GBA* mutation, and stratified them according to severity of the gene variants. These Authors found that severe variants (L444P, D409H, W291X, H255Q, and R131L) were associated with higher risk for iRBD compared to the mild N370S variant. Additionally, there was a trend for severe variants to drive towards faster conversion to a neurodegenerative disease [29••]. However, the number of severe variant carriers was very low and further studies are needed to confirm this finding.

Recently, mutations in the *TMEM175* gene have gained academic attention for their relationship with PD risk [74]. Krohn and colleagues have identified the p.M393T variant on *TMEM175* as strongly associated with the risk of both PD and iRBD [75]. The p.Q65P variant was then associated with an increased rate of phenoconversion to a synucleinopathy [31••]. Recent cross-sectional studies on large cohorts have also detected genetic associations between iRBD risk and variants in the genes *SNCA*, *BST1,* and *LAMP3*, which could further expand our knowledge on the links between genetics and development and progression of iRBD [76, 77].

Epigenetic mechanisms have also been recently studied in relation to their progression risk from iRBD to neurodegeneration. In a recent small, preliminary study on 78 patients with iRBD of which 16 converted to PD after 3.75 years, hypomethylation at the Cytosine-phosphate-Guanine (CpG) 17 of the *SNCA* intron 1 has been associated with increased risk of clinical phenoconversion, and hypomethylation to the CpG 14, 15, and 16 was associated with progression of iRBD symptoms [32•].

## Neurophysiological Markers of Neurodegeneration

Electrophysiology is an essential tool to diagnose and characterize iRBD and has long been employed to identify changes in sleep structure and brain electrical activity with potential to predict evolution of iRBD into a neurodegenerative disease. Various patterns of REM and non-REM sleep, and wake activity have been studied in relation to disease severity and progression, and to their prediction of conversion to a synucleinopathy [78].

### REM Sleep without Atonia

The finding of an abnormal electromyographic activity on PSG during REM sleep is denominated REM Sleep Without Atonia (RSWA) and is a pathognomonic feature of RBD. According to its characteristics, RSWA can be tonic, or phasic. The severity of RSWA in iRBD increases over time and this arguably reflects the progression of the brainstem damage induced by the neurodegenerative process [79]. The percentage of tonic RSWA at baseline, in iRBD, has been established as a strong predictor of future conversion to PD [33].

Tonic and phasic RSWA are thought to represent the electrophysiological expression of different pathophysiological alterations taking place in the brainstem [80, 81]. Recent studies have focused on the possible different predictive role of either tonic or phasic RSWA towards neurodegeneration. One large study assessed 216 patients with iRBD who were followed-up for five years, and 26.9% of these iRBD patients developed a neurodegenerative disease [34•]. Baseline tonic RSWA showed a stable predictive capacity of future development of PD over time, whereas baseline phasic RSWA was only predictive of future conversion to DLB at long follow-up [34•]. This was confirmed in another recent study in which percentage of tonic RSWA was predictive of a more rapid conversion to parkinsonism, but not of cognitive impairment, thus suggesting that distinction between RSWA subtypes could predict future development of neurodegeneration in iRBD patients [35].

Isolated RSWA (iRSWA) is the detection of RSWA in absence of other symptoms ascribable to RBD [82]. It can be an incidental finding in up of 5% of PSG, and its frequency increases with age. iRSWA can be associated with other electrophysiological, clinical, imaging, or autonomic findings [16, 83, 36, 84]. A few studies have tested the hypothesis that iRSWA could represent an initial manifestation of neurodegeneration, yielding however conflicting results. Stefani and colleagues did not report any progression of iRSWA patients towards neurodegeneration after a 8.6-year follow-up [36], and in another study, iRSWA was not correlated with striatal dopamine levels as assessed with [^123^I]FP-CIT SPECT [37]. By contrast, Dede and colleagues, studying 67 iRSWA patients for at least 4 years, reported that 26.8% developed RBD and 8.9% developed a neurodegenerative disorder. This study, however, lacked a control group to ascertain whether the progression was due to aging or by a genuine increased risk of iRSWA [38].

### Electroencephalography

Electroencephalography (EEG) studies in iRBD show a diffuse slowness of cortical activity [85], which correlates with cognitive tests exploring attention, executive functions, and verbal memory [86, 39]. Two longitudinal studies have assessed the predictive value of EEG alterations in iRBD towards neurodegeneration. One study enrolled 54 iRBD patients to perform quantitative EEG and to a 3.5-year follow-up. The iRBD patients who converted after follow-up showed higher δ and θ power in the cortex, with higher slow-to-fast power ratio. Most importantly, detection of diffuse cortical EEG slowing was predictive of conversion to DLB, whereas EEG slowing restricted to temporal and occipital lobes was predictive of conversion to PD [39]. In a second study on 121 patients with iRBD, of which 27 converted to either PD or DLB after four years, diffuse bursts of θ band together with a decrease of bursting in the α band could distinguish iRBD converters compared to controls [40].

EEG during non-REM sleep has also been studied as potential marker of neurodegeneration in iRBD. Cyclic Alternating Pattern (CAP) is a spontaneous, physiological rhythm of non-REM sleep composed of transient electro-cortical events of arousal, followed by retrieval to background EEG activity that is interpreted as an expression of arousal instability [87, 88]. The number and architecture of CAP is significantly altered in iRBD [89]. Melpignano and colleagues studied 67 iRBD patients and found that CAP cycles were longer, and their rate significantly decreased. In addition, they found that CAP rate was most reduced in those patients who converted earlier to a neurodegenerative disease [41]. Further, confirmatory studies will establish the potential of microstructural alterations of non-REM sleep architecture as potential markers of progression of neurodegeneration in iRBD.

### Fluid Biomarkers

Fluid biomarkers including cerebrospinal fluid (CSF) markers, such as oligomeric, total and phosphorylated α-synuclein, total and phosphorylated tau, amyloid-β_42_ and neurofilament light chain, have become increasingly investigated as a source of potential biomarkers providing insights into the pathogenesis of neurodegenerative diseases. PD patients with RBD have been shown to have higher CSF and serum levels of oligomeric α-synuclein compared to PD patients without RBD [90]. Furthermore, the presence of RBD in PD patients has shown to be a predictor of motor progression in patients with both low α-synuclein CSF levels and reduced striatal DAT [^123^I]FP-CIT uptake, and a predictor of cognitive decline in patients with low CSF levels of both α-synuclein and low amyloid-β_42_ [91]. A longitudinal study illustrated that lower baseline amyloid-β_42_ levels were predictive of cognitive decline at three-year follow-up only in PD patients with RBD [92]. Increased CSF prion protein levels have also been reported in PD patients with RBD compared to PD patients without RBD [93]. CSF inflammatory markers, including interleukin 1β and nitric oxide, as well as serum prostaglandin E2 have also been shown to be elevated in PD patients with RBD [90]. A recent study in probable iRBD patients illustrated that a reduced ratio of phosphorylated tau to total tau was associated with phenoconversion to a synucleinopathy disease at a 5-year follow-up highlighting the potential use of fluid biomarkers to track progression in iRBD [31••]. Future studies investigating fluid biomarkers in iRBD patients, prior to the clinically diagnosis of a synucleinopathy disease, are warranted to fully elucidate the potential utility of CSF and blood biomarkers to monitor the progression of RBD.

### Neuroimaging Biomarkers

The last decade has seen an increasing volume of neuroimaging studies, employing Positron Emission Tomography (PET), Single Photon Emission Computed Tomography (SPECT), Magnetic Resonance Imaging (MRI) and transcranial sonography techniques, to investigate the pathophysiology of iRBD and to help identify potential biomarkers to predict the progression of RBD and the conversion of iRBD to a synucleinopathy disease.

[^123^I]FP-CIT SPECT and transcranial sonography have been shown to detect subclinical changes in iRBD patients, similar to pathology seen in early PD [42–44]. Four longitudinal studies have used dopaminergic SPECT to investigate the progression of presynaptic striatal dopamine pathology as a biomarker in iRBD patients [42–45]. Iranzo and colleagues demonstrated that lower striatal presynaptic DAT availability, using [^123^I]FP-CIT SPECT, combined with hyperechogenicity of the substantia nigra, using transcranial sonography, had a predictive value of 100%, with 55% specificity, after 2.5 years to predict the conversion of iRBD patients to a neurodegenerative synucleinopathy [44]. Repeated [^123^I]FP-CIT SPECT scans show progressive loss of striatal DAT in iRBD patients over three years, with iRBD patients who converted to PD showing the greatest level of nigrostriatal dopaminergic dysfunction at baseline [42]. Furthermore, a reduction of [^123^I]FP-CIT SPECT greater than 25% in the putamen has been shown to discriminate iRBD patients, with DAT deficits, who converted to a synucleinopathy from iRBD patients who did not convert after three-year follow-up [43]. At a five-year follow-up [^123^I], FP-CIT SPECT had 75% sensitivity and 51% specificity to predict iRBD conversion to a synucleinopathy with a likelihood ratio of 1.54 [43]. Li and colleagues further highlighted the predictive value of decreased DAT in the putamen and striatum, using [^99m^TC]TRODAT-1 SPECT, in iRBD patients over 5 years with greater DAT deficits in those patients at high risk of progressing to a synucleinopathy [45]. Together these studies provide evidence to suggest that presynaptic nigrostriatal dopaminergic dysfunction, detected using SPECT imaging, could offer a valuable biomarker to monitor the progression of nigrostriatal deficits in RBD patients with the potential ability to aid the prediction of phenoconversion to a neurodegenerative synucleinopathy. DAT SPECT imaging is already used in clinical practice [94] therefore the platform to implement this tool in iRBD could be feasible. To aid potential translation into clinical practice, large, multicentre, longitudinal studies are warranted to further validate the use of SPECT imaging as a tool to predict phenoconversion in iRBD patients.

Glucose metabolism and perfusion changes have been reported in iRBD patients with spatial covariance analysis identifying abnormal PD-related metabolic brain networks [95, 96, 97, 98]. A longitudinal [^99m^Tc]ECD SPECT study illustrated increased perfusion in the hippocampus of RBD patients who developed a synucleinopathy at a 3-year follow-up compared to those who did not progress [46]. A logistical regression model combing increased PD-related covariance pattern expression with age was predictive of phenoconversion from iRBD to a synucleinopathy at an average follow-up of 4.6 years [47]. Furthermore, recent work by Kogan and colleagues supports the potential use of multiple [^18^F]FDG PET scans to measure progressive changes in PD-related brain pattern expression measures as a prodromal PD biomarker to predict phenoconversion in iRBD patients [48••].

Cardiac [^123^I]metaiodobenzylguanidine (MIBG) scintigraphy has also been investigated in iRBD patients. While cross-sectional studies have revealed abnormalities [99, 100, 101], a longitudinal study reported no changes in RBD patients at a 2.5-year follow-up [102]. These findings suggest that while sympathetic denervation may be abnormal in early RBD patients, [^123^I]MIBG might not be a sensitive biomarkers for the progression and phenoconversion in RBD patients.

Structural and functional MRI techniques have demonstrated changes in deep grey matter, cortical grey matter, microstructural white matter and disrupted functional connectivity networks in patients with RBD which can be associated with clinical symptoms [103]. Isolated RBD patients who converted to a clinically defined synucleinopathy, at a three-year follow-up, showed greater cortical thinning in frontal, parietal and occipital cortices compared to iRBD patients who did not convert [49••]. Pereira and colleagues reported cortical thinning as a predictor of phenoconversion in iRBD [49••]. Furthermore, grey matter atrophy in the inferior frontal gyrus has been associated with phenoconversion at 5-year follow-up [31••]. Together these studies suggest that structural neuroimaging could act as a predictive biomarker for increased risk of progression to a synucleinopathy.

Diffusion-weighted MRI has been employed to define longitudinal brain connectome progression scores, using interpretable machine learning algorithm, to evaluate the progression patterns in iRBD patients as a prodromal phase of PD [104•]. The longitudinal connectome progression pattern in iRBD patients was similar to that of de novo PD patients, highlighting the potential of this tool as a biomarker for the neurodegenerative prodromal phase of synucleinopathies [104•]. This study highlights the potential future use of MRI-based computational biomarkers to predict the progression and conversion of RBD with high sensitivity and specificity. Longitudinal studies, such as the Oxford PD Centre Discovery Cohort MRI substudy (OPDC-MRI) [105], are ongoing to validate the use of structural and functional MRI techniques, combined with clinical data, as biomarkers to predict the progression and phenoconversion of iRBD to synucleinopathies.

### Conclusion

**Fig. 1:**
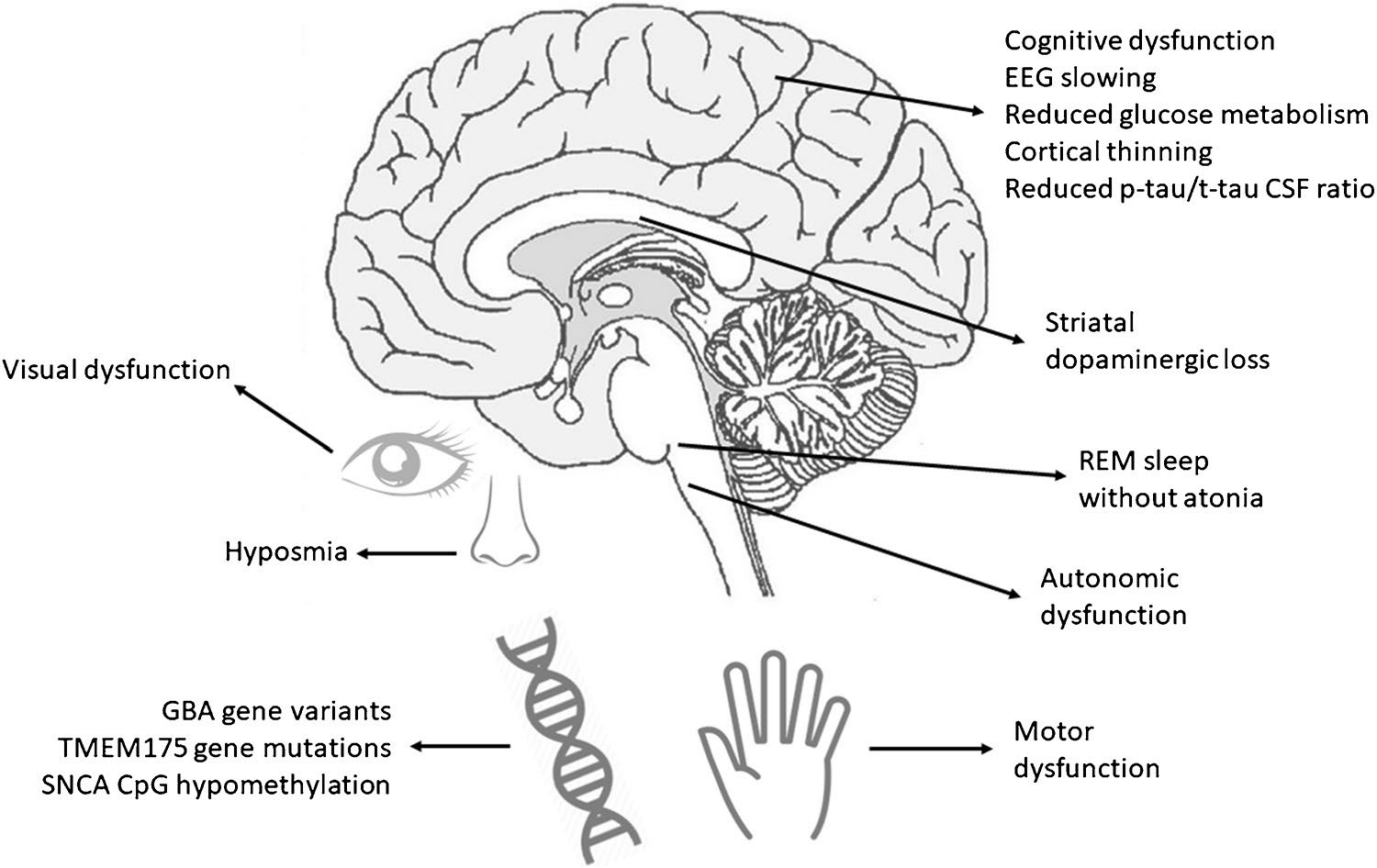
Schematic summary of the main areas of biological alterations found in association with increased rate of progression and/or phenoconversion of iRBD patients to synucleinopathy. Abbreviations: EEG: electroencephalogram; GBA: glucocerebrosidase; REM: rapid eye movements; SNCA: synuclein; TMEM175: transmembrane protein 175

As a high-risk population for conversion to synucleinopathies, iRBD offers a valuable therapeutic window for application of early neuroprotective interventions and disease-modifying therapies. Recent longitudinal studies have highlighted a variety of potential biomarkers, including clinical, neurophysiological, genetic, CSF, serum, and neuroimaging, for monitoring disease progression and predicting iRBD conversion into synucleinopathies (Fig. 1). However, the role of biomarkers as predictors of iRBD remains to be fully elucidated. A combined multimodal biomarker model could offer a sensitive and specific tool to predict the progression of RBD and conversion to synucleinopathies. Future studies are required, most notably large, multicentre, longitudinal studies, to validate these potential biomarkers and step towards their use as endpoints in future clinical trials.
